# Characterization of the Endometrial Microbiota of Healthy Mares Across the Estrous Cycle

**DOI:** 10.3390/ani16040618

**Published:** 2026-02-15

**Authors:** Gian Guido Donato, Denis Necchi, Fabrizia Gionechetti, Ugo Ala, Patrizia Nebbia, Patrizia Robino, Maria Cristina Stella, Hilde Vandaele, Alberto Pallavicini, Tiziana Nervo

**Affiliations:** 1 Department of Veterinary Sciences, University of Turin, 10095 Grugliasco, Italy; gianguido.donato@unito.it (G.G.D.);; 2 Keros Embryo Transfer Center, 8980 Passendale, Belgium; 3 Department of Life Sciences, University of Trieste, 34127 Trieste, Italy

**Keywords:** horse, mare, microbiota, estrous cycle, uterus, endometrium

## Abstract

In the past, bacteria detected in the mare’s uterus were generally interpreted as a sign of endometritis, since the uterus was considered a sterile environment. This assumption has been challenged by the introduction of culture-independent molecular techniques, particularly 16S rRNA amplicon sequencing, which have demonstrated that healthy mares harbor an endometrial microbiota. The aim of this study was to characterize the endometrial microbiota of healthy mares and to determine whether microbial composition differs between estrus and diestrus. Endometrial samples were collected from eleven healthy Standardbred mares during estrus and diestrus and analyzed by sequencing the V1–V2 region of the bacterial 16S rRNA gene. A total of 24 bacterial phyla and 599 genera were identified. At the phylum level, Firmicutes, Proteobacteria, Bacteroidota, and Actinobacteriota accounted for most of the relative abundance, while the most abundant genera were *Staphylococcus*, *Acinetobacter*, *Sphingomonas*, *Corynebacterium*, *Streptococcus*, *Clostridium*, and *Pseudomonas*. Alpha diversity was significantly higher during estrus, likely due to hormonally mediated changes in cervical opening and local immunity. Beta diversity analyses showed substantial overlap between estrus and diestrus samples. The phase of the cycle had a weak effect on microbiota structure, while inter-individual differences between mares explained a larger proportion of the observed variation. These findings suggest that the uterine microbiota of healthy mares is largely stable across the estrous cycle, with phase-dependent and mare-specific fluctuations in microbial composition.

## Simple Summary

Traditionally, the presence of bacteria in the mare’s uterus was interpreted as evidence of endometritis, as the uterine environment was long considered sterile. This assumption has been challenged by the introduction of culture-independent molecular techniques, particularly 16S rRNA amplicon sequencing, which have demonstrated that healthy mares harbor an endometrial microbiota. How this microbial community changes during the reproductive cycle, however, is still not well understood. The aim of this study was to describe the uterine microbiota of healthy mares and evaluate whether it differs between estrus and diestrus. Samples were collected from the same mares during both phases and analyzed through a 16S rRNA amplicon sequencing technique. The most abundant genera were *Staphylococcus*, *Acinetobacter*, *Sphingomonas*, *Corynebacterium*, *Streptococcus*, *Clostridium* and *Pseudomonas.* Bacterial diversity was higher during estrus, while the overall structure of the microbiota remained largely stable across the cycle with phase-dependent and mare-specific fluctuations. These results provide new insight into the uterine microbiome of healthy mares.

## 1. Introduction

For decades, the mare’s uterus was considered a sterile environment, and the detection of bacteria in uterine samples was commonly interpreted as an indication of endometritis [1]. This assumption has been challenged by the introduction of culture-independent molecular techniques, particularly 16S rRNA amplicon sequencing, which have demonstrated that clinically healthy mares harbor an endometrial microbiota [2,3].

The uterine environment undergoes marked physiological changes throughout the estrous cycle. During estrus, increasing estrogen concentrations promote endometrial edema, glandular secretions, and increased vascular permeability, while also softening and relaxing the cervix, thereby allowing communication between the vagina, cervix, and uterus [4]. Diestrus begins at the time of ovulation with the formation of the corpus luteum, which produces progesterone, the dominant hormone of this phase. Diestrus is characterized by the presence of a corpus luteum, firm uterine tone, absence of edema, and cervical closure [4,5], forming an effective barrier against ascending contamination. Furthermore, during estrus, when estrogen predominates, the uterine immune response is thought to be more efficient and capable of clearing infections compared to the progesterone-dominated diestrus phase, when physical clearance is reduced and the susceptibility to uterine infection increases [6,7,8,9].

Studies in humans and various other species have shown that microbial communities within the reproductive tract and gut shift across the sexual cycle, mediated by sexual hormones [10,11,12,13,14]. To date, research investigating these dynamics in the mare is still limited. Heil et al. [15] identified differences in the uterine microbiota between anestrus and estrus, observing greater bacterial diversity, richness, and abundance during anestrus. Conversely, Barba et al. [16] detected no significant variation in the vaginal microbiota between estrus and diestrus. These discrepancies highlight the need for further investigation into how physiological changes throughout the cycle influence the endometrial microbiota in the mare.

The aim of the present study was to characterize the endometrial microbiota of healthy mares and to determine whether microbial composition differs between estrus and diestrus.

## 2. Materials and Methods

### 2.1. Animals and Experimental Design

Eleven clinically healthy Standardbred mares aged 5–15 years (mean ± SD: 9.6 ± 3.9) were included in the study during the 2022 breeding season. Written informed consent was obtained from the owner of the animals involved in this study. All mares were pluriparous (mean ± SD: 2.6 ± 1.2 foalings) and used as embryo recipients at the Keros Embryo Transfer Center (Passendale, Belgium). Sampling took place at least nine months after the mares’ last foaling. The mares had been housed at the facility for >3 months before the start of the study, kept in group stalls, and fed hay and corn silage, with free access to water.

Only mares without a history of reproductive disorders were considered for enrollment. A complete breeding soundness evaluation was performed to confirm reproductive normality and the absence of clinical signs of endometritis. In addition, a double-guarded uterine swab was obtained from each mare for bacteriological culture, and only those with negative results were included. No “a priori sample” size was calculated; the sample size was based on the availability of clinically healthy mares meeting the inclusion criteria.

Transrectal ultrasonography was carried out every other day to monitor the estrous cycle and determine ovulation. Estrus samples were collected when the following criteria were simultaneously present: a dominant follicle > 35 mm, uterine edema graded ≥ 3/5 (subjective scale from 0 = no edema to 5 = pronounced edema [17]), and a relaxed, open cervix. Diestrus samples were collected 6–9 days after ovulation. When a well-defined corpus luteum was visible, uterine edema was absent (0/5), uterine tone was increased, and the cervix was tightly closed.

For each mare, one endometrial sample was obtained during the follicular phase and a second sample during the luteal phase of the same estrous cycle. When diestrus was the first phase sampled, the preceding estrus was monitored to determine the ovulation date.

The procedures performed are routine non-invasive clinical practices. During the whole study adverse events were not detected. This study did not have humane endpoints. The order of sample collection (estrus first or diestrus first) was randomized using the RAND function of Microsoft Excel.

No protocol registration was performed.

### 2.2. Sample Collection

The perineum was washed three times with water and povidone–iodine soap (Betadine; MEDA PHARMA SpA, Milan, Italy) and then dried with a paper towel. Using a sterile glove, endometrial samples for microbiota analysis and bacteriological culture were collected with a double-guarded cytobrush and a double-guarded swab, respectively (Minitube GmbH, Tiefenbach, Germany), as previously described [18]. Briefly, the device was inserted into the vagina and advanced up to the internal cervical os, where the inner sheath was extended. Once the tip reached the uterine body, the brush or swab was exposed and gently rotated against the endometrial surface for approximately 30 s. After sampling, the tip was retracted back into the protective outer sheath before the instrument was removed. The cytobrush for microbiota analysis was collected first, followed by the swab for bacteriological culture. Eleven swab samples underwent bacteriological analysis as previously described [19]. As no bacterial growth was detected in any uterine sample upon culture, all animals were confirmed for inclusion in the study. 

For microbiota analysis, the cytobrush tip was aseptically cut with sterile scissors and placed into a sterile 2 mL Eppendorf tube (Sigma-Aldrich, St. Louis, MO, USA). Samples were kept at 4 °C during transport to the laboratory, which occurred within a maximum of two hours, and were subsequently stored at −20 °C for up to one month until processing.

### 2.3. 16S rRNA Gene Sequencing

Sequencing of the bacterial 16S rRNA gene was performed on 22 uterine brushes. DNA extraction from the frozen and thawed brushes was performed using the E.Z.N.A.^®^ Soil DNA Kit (Omega Bio-Tech, Norcross, GA, USA) as previously reported [20]. Extraction blanks (laboratory reagents only) were included as negative controls to assess possible contamination from the extraction kit. Furthermore, two sterile unused brushes served as negative controls to check for possible contamination from the sampling device. DNA was quantified using the NanoDrop 2000 spectrophotometer (Thermo Fisher Scientific, Waltham, MA, USA). The 16S V1–V2 region was chosen because it minimizes off-target amplification of host mitochondrial DNA in low-biomass samples, due to its reduced sequence similarity to the mammalian mitochondrial genome [21]. V1–V2 primers were tailed with i5 and i7 Nextera adapters allowing barcoding with a second amplification step. PCR was performed in a 25 µL volume reaction containing 12.5 µL Accustart II PCR ToughMix 2X (Quanta Bio, Beverly, CA, USA), 1.25 µL EvaGreen™ 20X (Biotium, Fremont, CA, USA), 1 µL 16S-i5-XT-27F primer (5′-TCG TCG GCA GCG TCA GAT GTG TAT AAG AGA CAG AGV GTT YGA TYM TGG CTC AG, 10 µM), 1 µL 16S-i7-XT-338R primer (5′-GTC TCG TGG GCT CGG AGA TGT GTA TAA GAG ACA GTG CTG CCT CCC GTA GGA GT, 10 µM) and 2 µL (50 ng) DNA template. PCR was performed in a CFX 96™ PCR System (Bio-Rad Laboratories Inc., Hercules, CA, USA) with a limited number of cycles monitored in real time: 94 °C for 30 s, 55 °C for 20 s, 72 °C for 30 s and a final extension of 72 °C for 5 min. All amplicons were checked for quality and size by running 2 µL on 2% agarose gel electrophoresis and visualized a ≈ 350 bp band. They were then sent to an external laboratory (BMR Genomics, Padua, Italy) for barcoding, and sequencing using the Illumina MiSeq platform (Illumina, San Diego, CA, USA).

### 2.4. Bioinformatic and Statistical Analysis

Sequencing data were initially processed and analyzed using CLC Microbial Genomics Module version 23.0.3 (QIAGEN Bioinformatics, Hilden, Germany). Raw reads were quality filtered to remove adapters and low-quality sequences (quality score threshold: 0.03). Paired-end reads were merged and primers were trimmed. Paired-end reads were denoised into amplicon sequence variants (ASVs) using the DADA2 algorithm [22] implemented in CLC Microbial Genomics. ASVs were taxonomically classified using the SILVA database version 138.1 with a confidence threshold of 97%. All sequences of non-bacterial origin (i.e., chloroplasts and sequences not assigned to the bacteria kingdom) were removed.

Further analyses were conducted in R version 4.2.2 (Vienna, Austria) using the packages phyloseq, vegan, ggplot2, microbiome, and DESeq2. Data were rarefied to the minimum library size for alpha- and beta-diversity analyses. Alpha diversity was calculated using observed richness (Observed ASVs), Chao1, Shannon, and Simpson indices and compared between phases using a paired Wilcoxon signed-rank test. Beta diversity was estimated using Bray–Curtis dissimilarity. Differences between estrus and diestrus were assessed by permutational multivariate analysis of variance (PERMANOVA). To account for the paired study design, permutations were constrained within individual mares by including mare identity as a blocking factor. In addition, a marginal PERMANOVA model including both mare identity and cycle phase was used to estimate the proportion of variance explained by each factor independently. Homogeneity of multivariate dispersions was evaluated using the betadisper function. Differential abundance analysis at the genus level was performed using DESeq2 on non-rarefied count data (DESeq2 internal normalization). Statistical significance was set at *p* < 0.05.

## 3. Results

**Figure 1:**
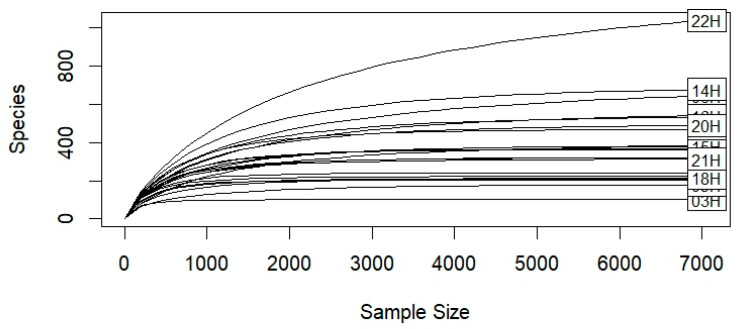
Taxonomy rarefaction curves for each of the 22 endometrial samples. The sequencing depth is sufficient for subsequent analyses.

A total of 2,581,412 paired-end reads were obtained. After filtering, 2,187,428 reads remained, resulting in 24,388 ASVs. Rarefaction curves indicated that sequencing depth was sufficient to observe the total diversity present in the microbial community (Figure 1).

### 3.1. Relative Abundance

A total of 20 bacterial phyla were observed in diestrus and 23 during estrus. Twenty-four phyla in total were identified (Supplementary Materials Figure S1). The most abundant phylum overall was Firmicutes (D: 39.2%, E: 38.8%), followed by Proteobacteria (D: 29.8% E: 22.7%), Bacteroidota (D: 14.0%, E: 16.6%) and Actinobacteriota (D: 12.1%, E: 16.2%). These phyla accounted for nearly 95% of relative abundance, while the remaining phyla each showed a relative abundance < 1.5%.

**Figure 2:**
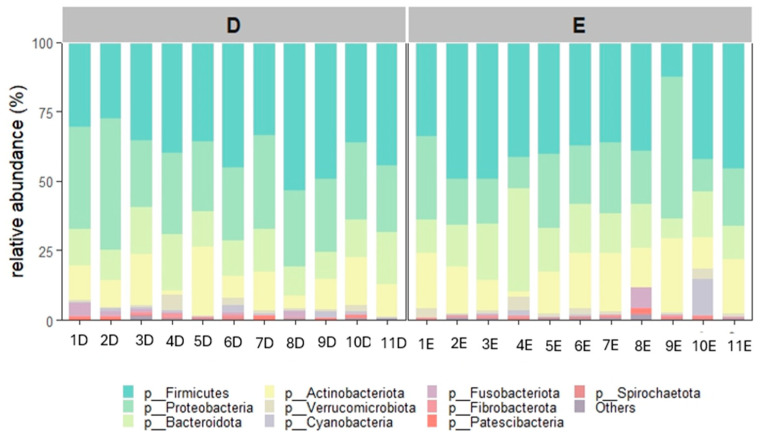
Relative abundance of the 10 most abundant phyla detected in the 11 mares during diestrus (D) and estrus (E).

**Figure 3:**
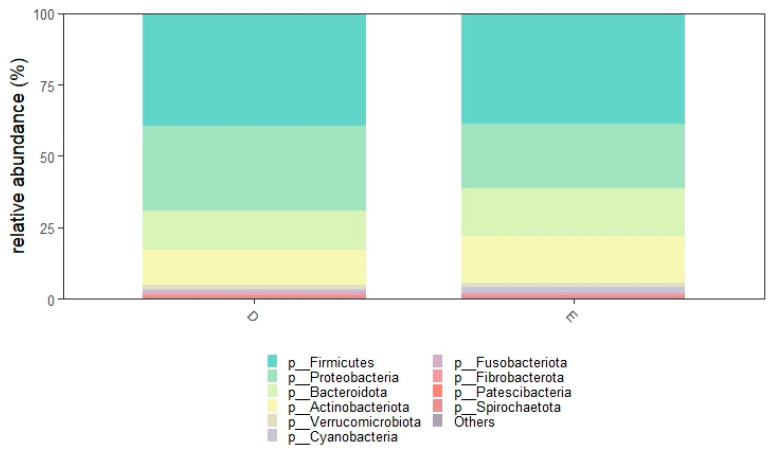
Relative abundance of the 10 most abundant phyla grouped according to cycle phase. Diestrus (D) and estrus (E).

Relative abundances at the phylum level are shown in Figure 2 and Figure 3. Relative abundances of all the 24 phyla are shown in the Supplementary Materials (S2).

At the genus level, 599 different genera were observed, 392 during diestrus and 528 during estrus (Supplementary Materials Figure S3). The most abundant taxa was *Staphylococcus* (D: 7.8%, E: 8.2%), followed by *Acinetobacter* (D: 7.2%, E: 5.3%), *Sphingomonas* (D: 6.8%, E: 4.7%), *Corynebacterium* (D: 4.6%, E: 6.6%), *Streptococcus* (D: 4.4%, E: 6.6%), *Rikenellaceae RC9 gut group* (D: 3.0%, E: 4.2%), *Clostridium* (D: 2.0%, E: 4.2%,), *Pseudomonas* (D: 2.2%, E: 3.1%,). Furthermore, *Porphyromonas* accounted for 1.9% and 1.0% in diestrus and estrus, respectively, while *Escherichia-Shigella* accounted for 0.8% and 0.4%.

**Figure 4:**
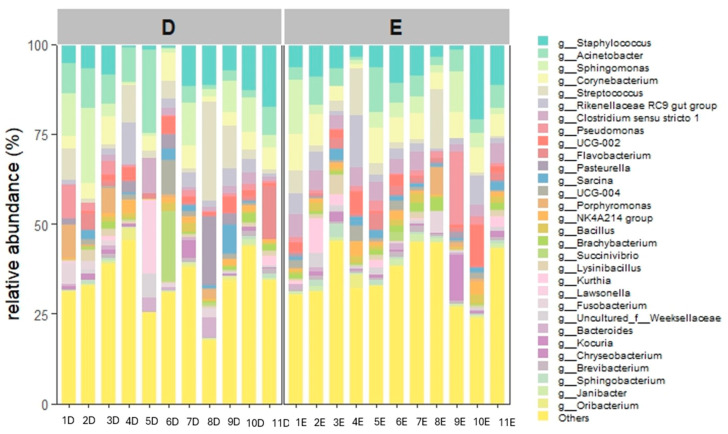
Relative abundance of the 30 most abundant genera detected in the 11 mares (22 samples) during diestrus (D) and estrus (E). This plot highlights the marked inter-individual heterogenicity.

**Figure 5:**
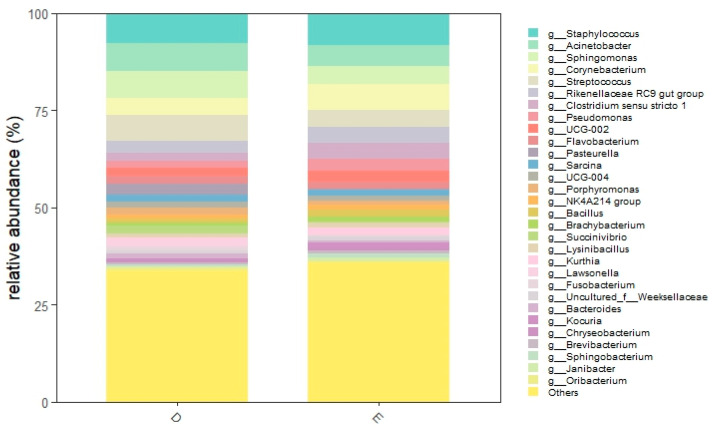
Relative abundance of the 30 most abundant genera detected grouped according to cycle phase. Diestrus (D) and estrus (E).

Relative abundances at the genus level are shown in Figure 4 and Figure 5. Relative abundances of the 40 most abundant genera are shown in the Supplementary Materials (S4).

### 3.2. Alpha and Beta Diversity

**Figure 6:**
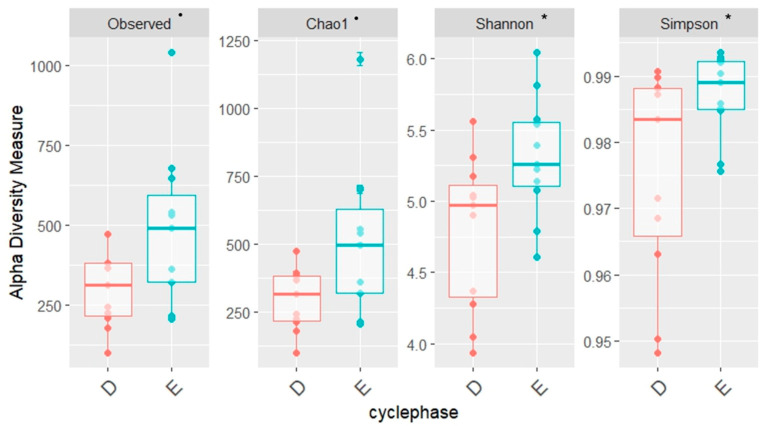
Variation in endometrial microbial alpha diversity based on the cycle phase (diestrus—D—red and estrus—E—blue) assessed by observed richness, Shannon, Simpson, and Chao 1. * indicates a *p*-value <0.05, while ^•^ indicates 0.1 < *p* < 0.05. Statistically significant differences between estrus (E) and diestrus (D) were observed according to the Shannon and Simpson indices.

An increase in alpha diversity was observed during the follicular phase, as described through Shannon (*p* = 0.023) and Simpson indices (*p* = 0.047). For observed richness and Chao 1, *p*-values were 0.065 (Figure 6).

**Figure 7:**
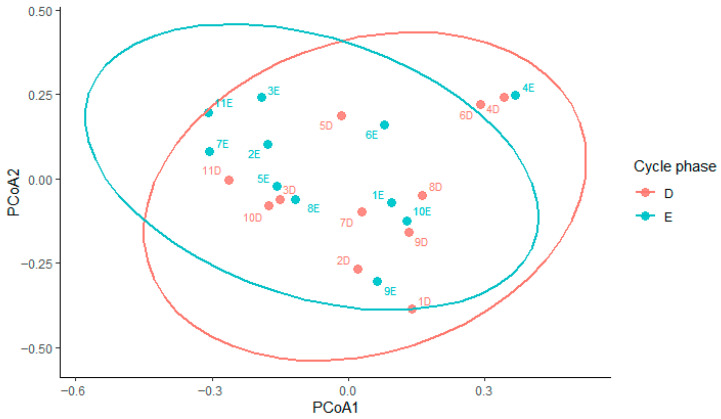
Two-dimensional PCoA plot based on Bray–Curtis dissimilarity, showing samples collected during diestrus (D) and estrus (E). Samples from the two cycle phases show overlapping clustering.

Beta diversity based on Bray–Curtis dissimilarity showed substantial overlap between estrus and diestrus samples (Figure 7). Bray–Curtis-based PERMANOVA with mare as a blocking factor showed a weak effect of cycle phase on overall community structure (R^2^ = 0.05, *p* = 0.054), indicating subtle differences between estrus and diestrus that did not result in clear separation of samples. In contrast, marginal PERMANOVA showed that mare identity explained a much larger proportion of the variance in beta diversity (R^2^ = 0.52, *p* = 0.002), highlighting pronounced inter-individual variability. Homogeneity of dispersion did not differ between phases (betadisper, *p* = 0.35).

### 3.3. Differential Abundance

At the genus level, Oceanobacillus was more abundant during estrus, while Helcococcus and Actinobacillus showed higher abundance during diestrus (Supplementary Materials Figure S5).

## 4. Discussion

In this study, we characterized the endometrial microbiota of healthy mares and explored how it changes between estrus and diestrus, revealing differences in diversity across the cycle.

We identified 24 different phyla and 599 different genera. A higher number of phyla and genera was detected during estrus. The uterine microbiota of healthy mares was composed mainly of Firmicutes, with Proteobacteria, Bacteroidota and Actinobacteriota following in decreasing order. Together, these four phyla represented more than 95% of the total community. A similar distribution has been reported by several authors [8,16,23,24] who also found Firmicutes to be the predominant phylum in both the uterus and vagina of healthy mares. Other studies [2,15,25], however, described a different pattern, with Proteobacteria being more abundant than Firmicutes. It is worth noting that in the study by Holyoak et al. [2], who compared healthy mares from different geographical locations, Firmicutes was the most abundant phylum only in samples from one specific location. This finding clearly shows how challenging it is to compare uterine microbiota studies carried out in different environments or under different management conditions. Even when the sampling technique and sequencing approach are similar, the microbial profile can change substantially.

At the genus level, the most abundant genera were *Staphylococcus, Acinetobacter, Sphingomonas, Corynebacterium, Streptococcus, Rikenellaceae RC9 gut group, Clostridium, Pseudomonas* and *Porphyromonas*. *Escherichia-Shigella* had a relative abundance of less than 1%. This result is particularly interesting because the most abundant genera colonizing the uterus of healthy mares comprise bacterial species that are considered in the clinical literature to be common pathogens causing endometritis in horses, such as *Staphylococcus*, *Streptococcus* and *Pseudomonas* [9,26]. Our findings are supported by the study of Holyoak et al. and Thomson et al. [2,25], who identified *Pseudomonas*, *Escherichia-Shigella*, *Streptococcus*, and *Staphylococcus* among the most abundant genera in the uterus of healthy mares. Therefore, these genera are probably not always responsible for endometritis but are normal constituents of the uterine microbiome of healthy mares that can cause endometritis and colonize the uterus under specific pathological conditions [8]. Differences between studies at the genus level were also evident: Heil et al. [26] described *Klebsiella*, *Mycoplasma*, *Aeromonas* and *Citrobacter* as the most abundant genera, accounting for more than half of the relative abundance of the endometrial microbiota, whereas these taxa were absent or detected only at very low abundance in our samples. Similarly, Guo et al. [27] identified *Burkholderia* and *Chlamydia* as predominant genera, which were either rare or not detected in our study. Such differences are likely related to differences in mare populations, management systems, diet, environment, reproductive status, and technical aspects of sample processing such as DNA extraction protocols and the targeted 16S rRNA regions [28,29,30].

Overall, our data suggest that the endometrial microbiota of clinically healthy mares varies across the estrous cycle, with higher alpha diversity during estrus. Cycle-associated microbiome variation has been described in women and other species [12,13,14,31,32,33,34]. The changes we observed in the endometrial microbiome could be mainly explained by two factors. The first one is hormonal fluctuation during the estrous cycle, a well-known factor that influences the overall structure and function of the reproductive tract microbiome [14,35]. In the mare, steroid hormones are also known to modulate the effectiveness of the uterine immune response, further contributing to these cycle-related shifts [6,7,36,37]. The second is the presence of the cervix: this dynamic organ acts as a barrier between the vagina and uterus to prevent the entry of infectious agents during diestrus and pregnancy [38,39]. During estrus, however, the cervix relaxes and opens, allowing the entrance of semen and elimination of mucus, excess spermatozoa, and contaminants introduced by breeding [40]. Therefore, if ascension of bacteria through the cervix is a major determinant of the uterine microbiome, this could explain the observation that diversity increases during estrus, when cervical relaxation allows communication with the vagina, an environment that harbors a higher microbial biomass than the uterus [14,41,42]. However, it should be acknowledged that these interpretations, although biologically plausible, are speculative, as they are based on previously described physiological mechanisms rather than on direct measurements.

These observations should be considered alongside previous studies that have examined different regions of the reproductive tract. Barba et al. [16] described the vaginal microbiota of Arabian mares in estrus and diestrus and found no statistically significant differences in alpha or beta diversity, concluding that the vaginal microbiota is stable during the estrus cycle. However, this work investigated the vaginal microbiome, which is not dependent on cervical opening and uterine immune response, factors that could explain variations throughout the cycle. Furthermore, a recent study by Heil et al. [15] investigated the differences between the uterine microbiota in estrus and anestrus and found lower diversity and richness during estrus and a different microbial composition between the two phases. They attributed this difference to the variance in immune responsiveness of the endometrium, which is under the influence of estrogens in the follicular phase, enabling a strong immune response and possibly a less diverse microbiome. These results are, however, difficult to compare directly with ours because the studies examined different reproductive phases (anestrus versus diestrus) and because the interval between sampling points was considerably shorter, maximum two weeks in our study, as opposed to several months in the study by Heil et al., which collected the samples during the breeding season and the subsequent non-breeding season.

Beta diversity analyses showed a substantial overlap between samples collected during estrus and diestrus. PERMANOVA indicated only a weak effect of cycle phase, while mare identity accounted for a larger proportion of the observed variation. This suggests that inter-individual differences between mares outweigh the effects of cycle phase on beta diversity, even under standardized housing conditions, as also reported in other studies of the equine uterine microbiome [23]. Such pronounced individual variability appears to be a characteristic of the equine uterine microbiota. This feature potentially constraining broad cycle-based interpretations thereby hindering straightforward comparisons across studies. Together, these findings support the presence of a core endometrial microbiota that remains largely stable across the estrous cycle, accompanied by mare-specific and phase-dependent shifts. However, the relatively small number of mares included in this study reduces the statistical power of the analyses; therefore, the cycle-related patterns observed should be considered exploratory. Nevertheless, our results contribute to improving current knowledge of the uterine microbiota in healthy mares and emphasize the need for further studies to better distinguish normal physiological variation from microbiota alterations associated with reproductive disorders. In the future, this distinction may support the use of microbiota analysis as a complementary tool in the diagnosis and management of equine reproductive diseases.

## 5. Conclusions

This study shows that the endometrial microbiota of healthy mares varies across the estrous cycle. Although the overall structure remained largely stable, it was accompanied by phase-dependent and mare-specific fluctuations in microbial composition. Furthermore, microbial diversity was higher during estrus, likely due to hormonally mediated changes in cervical opening and local immunity.

## Data Availability

The data that support the findings of this study are available from the corresponding author upon reasonable request.

## Supplementary Materials

The following supporting information can be downloaded at https://www.mdpi.com/article/10.3390/ani16040618/s1, Figure S1: Number of phyla detected during estrus (E) and diestrus (D); S2: Relative abundance of the different phyla detected during diestrus (D) and estrus (E); Figure S3: Number of genera detected during estrus (E) and diestrus (D); S4: Relative abundance of the 40 most abundant genera detected during estrus (E) and diestrus (D); Figure S5: Differential abundance between estrus and diestrus at the genus level.
