# Supplementary figures and images for: Characterization of the Endometrial Microbiota of Healthy Mares Across the Estrous Cycle
Source: Animals (Basel). 2026 Feb 15;16(4):618. doi: 10.3390/ani16040618 (PMC12937484; doi:10.3390/ani16040618)

Figure S1: Number of phyla detected during estrus (E) and diestrus (D)

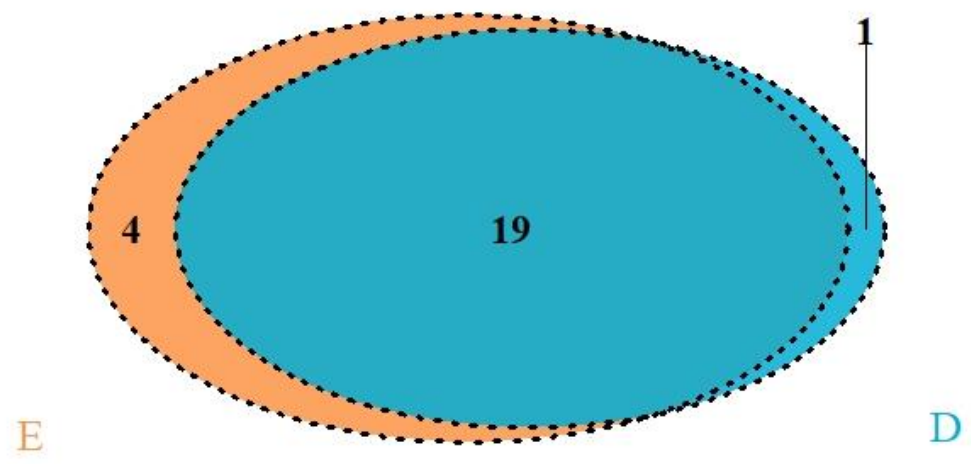

Supplement: Supplementary file 1 [file animals-16-00618-s001.zip › Figure S1.pdf]

Figure S3: Number of genera detected during estrus (E) and diestrus (D)

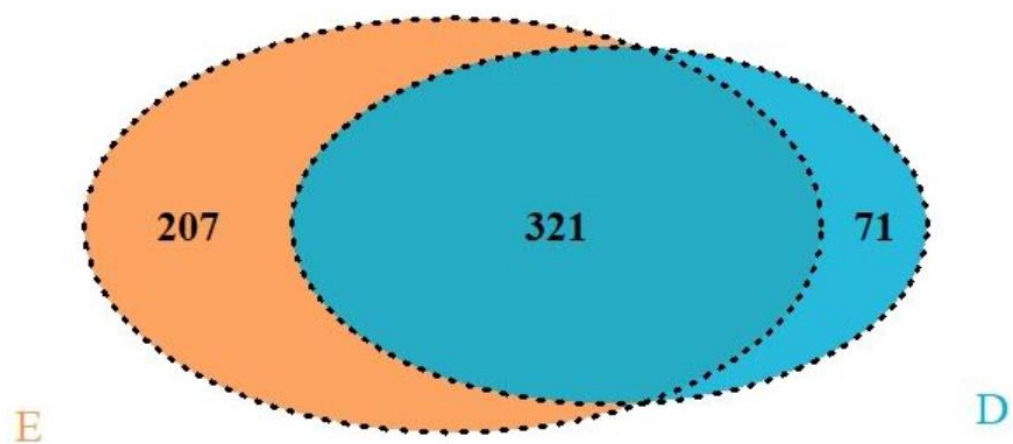

Supplement: Supplementary file 1 [file animals-16-00618-s001.zip › Figure S3.pdf]

Figure S5: Differential abundance between estrus and diestrus at the genus level

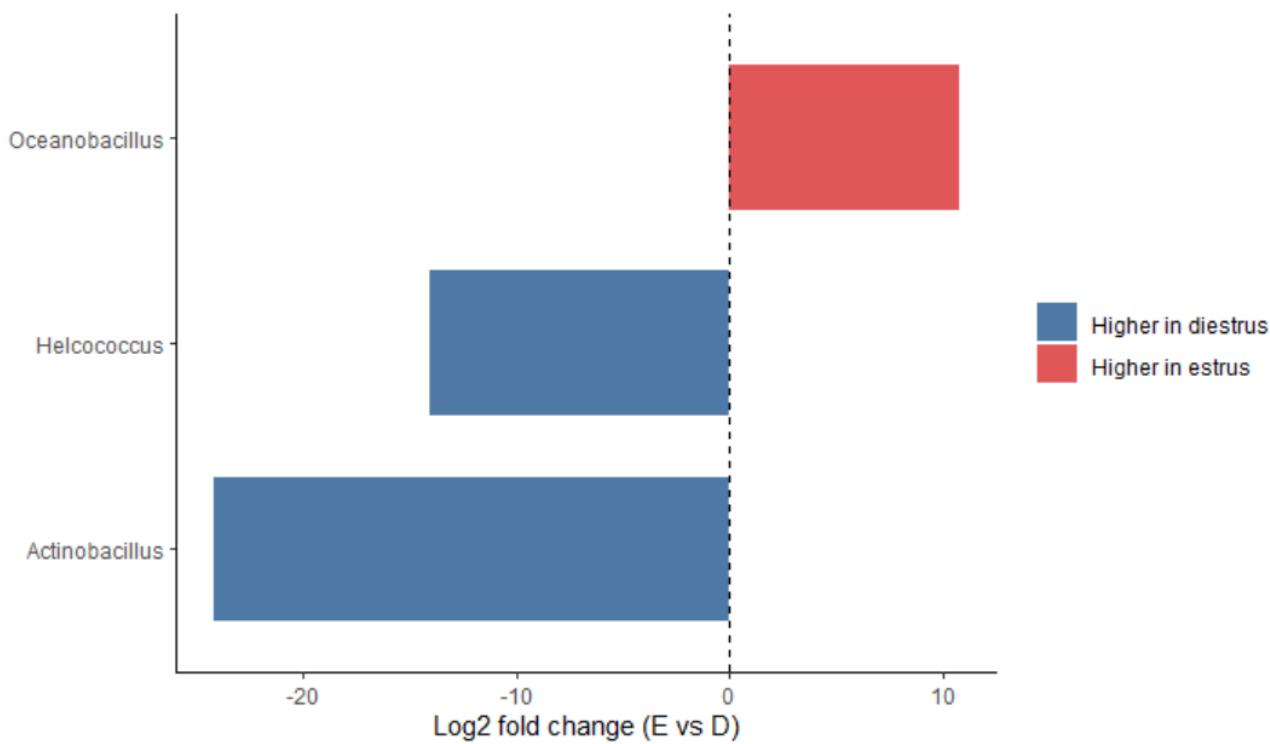

Supplement: Supplementary file 1 [file animals-16-00618-s001.zip › Figure S5.pdf]
